# Nurses' Experiences of Working With Healthcare Interpreters When Caring for Patients With Limited Proficiency in the Primary Language: A Qualitative Systematic Review

**DOI:** 10.1111/jocn.17740

**Published:** 2025-05-25

**Authors:** Marika Seremetkoska, Theresa Nielsen, Kathryn Farrell, Jenny Morris, Verily Thomas, Bronwyn Everett

**Affiliations:** ^1^ Western Sydney University Liverpool New South Wales Australia; ^2^ Oncology Bankstown Hospital, South Western Sydney Local Health District Liverpool New South Wales Australia; ^3^ Women's and Children's Health Bankstown Hospital, South Western Sydney Local Health District Liverpool New South Wales Australia; ^4^ Emergency Bankstown Hospital, South Western Sydney Local Health District Liverpool New South Wales Australia; ^5^ Infection Prevention and Control, Anglicare Baulkham Hills New South Wales Australia; ^6^ University of Wollongong, School of Nursing Wollongong New South Wales Australia

**Keywords:** bilingual health personnel, communication barriers, healthcare interpreting, interpreter services, language barriers, LEP, limited English proficiency, registered nurses, translating

## Abstract

**Background:**

Patients with limited proficiency in the primary language who do not receive a healthcare interpreter have poorer health outcomes. Clinician preference is an important factor in determining whether a healthcare interpreter will be used; yet little is known about their experiences and preferences for working with healthcare interpreters.

**Aims and Objectives:**

To review and synthesise the evidence examining nurses' experiences of working with healthcare interpreters when caring for patients with limited proficiency in the primary language.

**Methods:**

This review follows the reporting items contained within the ENTREQ statement. A systematic search of the literature was undertaken from inception to August 2024 in the databases CINAHL, Embase, Medline, PsychInfo, PubMed, Scopus, and ProQuest Dissertations and Theses Global. Included studies were assessed using the Critical Appraisal Skills Programme tool for qualitative studies. Thematic synthesis was undertaken on studies meeting the inclusion criteria, including studies undertaken in acute settings. Articles were limited to English‐language papers and peer‐reviewed.

**Findings:**

Eighteen studies representing the experiences of 416 nurses from eight countries were included in the review. Four themes were identified: (1) working with Interpreters, (2) tensions and challenges, (3) making connections, and (4) workarounds.

**Discussion:**

Nurses' experiences of working with healthcare interpreters were overwhelmingly positive; however, concerns about interpretation accuracy, unreliable technology, additional time required when working with a healthcare interpreter, and being sidelined were evident. Nurses commonly resorted to using bilingual nurse colleagues and family members when healthcare interpreters were unavailable.

**Conclusion:**

This review highlights the need for nurses to be allocated additional time in their patient loads when caring for patients with limited proficiency in the primary language. Healthcare policy that accommodates the use of bilingual healthcare professionals as well as the need for nurses to receive formal training on working collaboratively with healthcare interpreters is needed.

**Patient or Public Contribution:**

Not applicable due to systematic review.

**Summary**

- Caring for patients with limited proficiency in the primary language requires additional time to work with healthcare interpreters, which needs to be taken into account when allocating a patient load.
- Nurses make decisions on whether to use a healthcare interpreter based on the complexity and urgency of care and workflow.
- Nurses continue to use their bilingual colleagues as a first preference for patients with limited proficiency in the primary language.
- Despite the availability of technology, nurses still prefer face‐to‐face professional healthcare interpretation.

## 1 Introduction

Despite the improved health outcomes associated with using interpreters and increased satisfaction with communication expressed by patients, healthcare interpreters are consistently underutilised, with less than 30% of patients with limited proficiency in the primary language receiving an interpreter (Flores 2005; Karliner et al. 2007; Taira et al. 2019). Even in well‐resourced academic hospitals, clinician use of interpreters is highly variable. In a retrospective cohort analysis of all hospitalised patients admitted to a large academic centre in the United States, 66% of limited English proficiency (LEP) patients did not have an interpreter during their inpatient admission, despite the authors noting the hospital was well‐resourced (López et al. 2015). Similarly, an audit undertaken over four clinic days in a university hospital in the United Kingdom identified that out of 80 consultations requiring interpreters, only two patients received a trained interpreter (Madgulla et al. 2010).

Barriers to the use of interpreters are complex and not limited to lack of availability. Clinician preference is an important factor in determining whether a professional healthcare interpreter will be used, with some clinicians choosing to utilise bilingual staff to ‘get by’ (Chang et al. 2019). Patient preference has also been shown to influence the use of interpreters, with some patients preferring to use family members (Garrett et al. 2008).

Eliminating language barriers is essential in providing culturally competent, person‐centred nursing care (Ali and Johnson 2017) yet many nurses choose not to use interpreters, citing their use increases nursing workloads and complicates relationships between the patient and nurse (Eklöf et al. 2015). Given nurses are increasingly caring for people from culturally and linguistically diverse backgrounds who may have limited proficiency in the country's native language, this qualitative review aimed to synthesise nurses' experiences of and preferences for working with health care interpreters. In so doing, new and more comprehensive evidence of nurses' experiences across different populations and healthcare settings may be applied to improve the care of patients who experience language barriers.

## 2 Methods

### 2.1 Aim

The aim of this review was to synthesise qualitative studies describing nurses' experiences of and preferences for working with professional healthcare interpreters when communicating with patients with limited proficiency in the primary language. For this review, a professional healthcare interpreter is defined as an interpreter who has met and maintained standards set by educational institutions which qualify its graduates for professional practice (Healthcare Interpretation Network 2007) and is able to transfer complex, specialised messages in the health domain from a source language into a target language using spoken (or signed) language that accurately reflects the meaning (National Accreditation Authority for Translators and Interpreters 2021). The research question which guided this review was: What are nurses' experiences of and preferences for working with healthcare interpreters when communicating with patients with limited proficiency in the primary language?

### 2.2 Design

This qualitative review follows the reporting items contained within the Enhancing Transparency of Reporting the Synthesis of Qualitative research (ENTREQ) statement: methodology and methods, literature searching and selection, appraisal and synthesis of findings (Data S1) (Tong et al. 2012).

### 2.3 Inclusion and Exclusion Criteria

We included all primary studies that reported qualitative data and met the following inclusion criteria: (1) explored the experiences, perceptions, practices, or preferences of nurses when working with healthcare interpreters; (2) undertaken in adult, acute care settings; and (3) included direct quotations of nurses' experiences of or preferences for working with interpreters. Although this was a review of qualitative studies, initially no restrictions on study design were imposed to allow for mixed‐method studies that clearly reported qualitative findings. For the purpose of this review, qualitative findings were those that included direct quotations of nurses to ensure their voices could be heard and their experiences brought to life (Creswell 2014; White et al. 2014).

### 2.4 Data Sources and Search Strategy

Six databases (CINAHL, Cochrane, Medline, PubMed, Scopus and ProQuest Dissertations and Theses Global [PQDT]) were searched from inception to August 2024. A health librarian assisted with developing the search strategy with search terms and keywords used in combination with the Boolean operators ‘AND’ and ‘OR’ and proximity locators. Key terms included but were not limited to interpreter, communication barrier, language, limited English proficiency, and culturally and linguistically diverse (see Data S2).

A search of Google Scholar as well as backward searching of reference lists and forward searching of article citations using Scopus was undertaken to identify any additional studies (Atkinson et al. 2015). Articles were limited to English‐language papers and peer‐reviewed, with no restrictions on the language spoken by participants.

### 2.5 Study Screening

**FIGURE 1:**
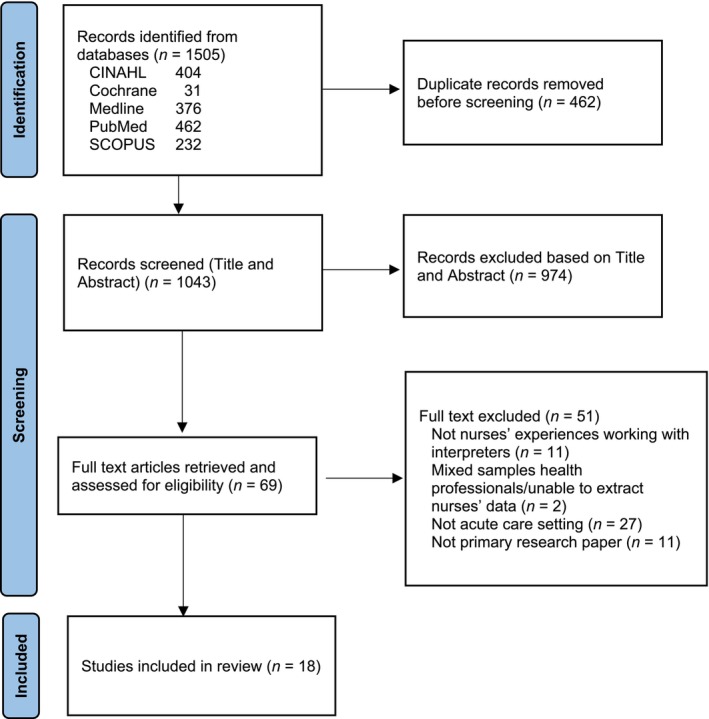
PRISMA flow diagram. [Colour figure can be viewed at wileyonlinelibrary.com]

An initial search identified 1505 potentially relevant studies (Figure 1) which included qualitative, quantitative, and mixed methods studies. Search results were imported into EndNote and duplicates were removed. Article titles and abstracts were independently screened for suitability for inclusion in the review by all authors. Disagreements were resolved through discussion with all team members until consensus was achieved. The full text of 69 articles was retrieved and examined for suitability for inclusion in the review, with 18 studies included in the final synthesis.

### 2.6 Quality Appraisal

Studies selected for inclusion were assessed for methodological quality using the Critical Appraisal Skills Programme (CASP) Qualitative Studies Checklist (Critical Appraisal Skills Programme (CASP) 2024). Assessment of methodological quality was independently undertaken by pairs of the review team (M.S. and J.M.; T.N. and K.F.; V.T. and B.E.) who then came together to resolve discrepancies. The quality assessment in this review was not undertaken to establish a quality threshold below which studies would be excluded but instead to gain an understanding of the relative strengths and weaknesses of the body of evidence and to take this into consideration during the process of synthesis (Centre for Reviews and Dissemination 2009). Overall, the quality of the studies included in this review was considered moderate, with most studies meeting 8 out of 10 criteria. Data were extracted to a summary table, which is presented in Table 1.

**TABLE 1:** CASP quality appraisal.

		1. Was there a clear statement of the aims of the research?	2. Is a qualitative methodology appropriate?	3. Was the research design appropriate to address the aims of the research?	4. Was the recruitment strategy appropriate to the aims of the research?	5. Was the data collected in a way that addressed the research issue?	6. Has the relationship between researcher and participants been adequately considered?	7. Have ethical issues been taken into consideration?	8. Was the data analysis sufficiently rigorous?	9. Is there a clear statement of findings?	10. How valuable is the research?
1	Ali and Watson 2018	Y	Y	Y	Y	Y	Y	Y	Y	Y	Y
2	Alkhaled et al. 2022	Y	Y	Y	Y	Y	Y	Y	Y	Y	Y
3	Cioffi 2003	Y	Y	Y	Unclear	Y	N	Y	Y	Y	Y
4	Coleman and Angosta 2016	Y	Y	Y	Y	Y	Y	Y	Y	Y	Y
5	Fatahi et al. 2010	Y	Y	Y	Y	Y	Unclear	Y	Y	Y	Y
6	Feiring and Westdahl 2020	Y	Y	Y	Y	Y	Unclear	Y	Y	Y	Y
7	Hemmila 2004	Y	Y	Y	Y	Y	Unclear	Y	Y	Y	Y
8	Høye and Severinsson 2008	Y	Y	Y	Y	Y	Unclear	Y	Y	Y	Y
9	Hsieh 2015	Y	Y	Y	Y	Y	Unclear	Y	Y	Y	Y
10	Keller and Carrascoza‐Bolanos 2023	Y	Y	Y	Y	Y	Y	Unclear	Y	Y	Y
11	Lee et al. 2018	Y	Y	Y	Unclear	Y	Unclear	N	Y	Y	Y
12	McCarthy et al. 2013	Y	Y	Unclear	Y	Y	Y	Y	N	Y	Y
13	Mottelson et al. 2018	Y	Y	Y	Y	Y	N	Y	N	Y	Y
14	Nailon 2006	Y	Y	Y	Unclear	Y	Unclear	Y	Y	Y	Y
15	Neiman 2019	Y	Y	Y	Y	Y	Unclear	Y	Y	Y	Y
16	Suarez et al. (2021a) (*Patient Education and Counseling*)	Y	Y	Y	Y	Y	N	Y	Y	Y	Y
17	Suarez et al. (2021b) (*Journal of Critical Care*)	Y	Y	Y	Y	Y	Unclear	Y	Y	Y	Y
18	Watts et al. 2018	Y	Y	Y	Y	Y	N	Y	Unclear	Y	Y

Abbreviations: N, No; Y, Yes.

### 2.7 Data Extraction and Synthesis

The results from all included studies were synthesised using thematic synthesis as described by Thomas and Harden (Thomas and Harden 2008). All studies were read in full, and participant quotations and text contained within the results sections were extracted. Line‐by‐line coding of the extracted findings was undertaken, and a preliminary coding framework developed to allow the translation of concepts from one study to another. Working in pairs, the study authors coded all extracts, with many categorised using several codes, and all authors came together to group related codes into descriptive themes.

## 3 Results

Table 2 reports the characteristics of the 18 studies included in this review. A total of 416 nurses were included in the studies, which were published between 2003 and 2023. Fifteen of the studies were qualitative designs, and three were mixed methods studies.

**TABLE 2:** Characteristics of included studies.

Author year	Country setting	Aim of study	Study design	Sampling and participants	Data collection and analysis	Main findings
Ali and Watson (2018)	UK: tertiary care hospital (NHS)	To explore nurses' perspectives of language barriers and their impact on the provision of care to patients with limited English proficiency from diverse linguistic backgrounds	Design: qualitative descriptive approach—	Purposive and snowball sampling. Participants: Registered Nurses (*n* = 59). Gender: Female (*n* = 32) Male (*n* = 27). Age: 25–52 years. Experience: 2–23 years. Ethnic background: not reported	Data collection: 26 individual interviews (50–75 min), 3 focus group discussions (75–90 min). Data collection period: not reported. Data analysis: thematic analysis	Difficult to arrange, particularly at night or out of hours and time consuming and expensive Difficult to use phone interpreter when patient has cognitive or hearing difficulties Increased challenges with communication when patients are distressed or post anaesthesia Delay in discharge due to not being able to book an interpreter and not being able to give home care instructions (increase LOS) Cancellation of appointments when no interpreter was available Issues where lack of medical terminology on behalf of interpreter leads to miscommunication Using an interpreter takes longer to communicate Patient ethnicity: Somalia, West Indies, Africa, Asia, Pakistan, India, Sri Lanka and Europe
Alkhaled et al. (2022)	Norway: a hospital in Norway	This qualitative study aimed to explore nurses' experiences in caring for patients with an ethnic minority background	Design: qualitative exploratory design	Purposive and snowball sampling. Participants: Registered Nurses (*n =* 21). Gender: Female (*n =* 19), Male (*n =* 2), Experience: 2–31 years. Ethnic background: (*n =* 4) Denmark, Germany, Cambodia, Chechnya	Data collection: 6 focus groups (60‐100min). Data collection period: between March and December 2021. Data analysis: thematic analysis	Three main themes, each with sub‐themes Caring for patients with an ethnic minority background was both challenging and enriching Due to language barriers, a lot more time and effort had to be spent communicating with the patients Simple conversations such as food choices or daily care could be informally conducted, but if medical issues were being discussed it was deemed better to call an interpreter Participants and other hospital staff with knowledge of additional languages could also be used as interpreters. However, this process could take up their time leading to poorer outcomes Some participants were happy to assist with interpreting, whilst others felt that this was not their job The use of gestures, pointing to body parts, drawings, pictures or videos were helpful stop gaps until an interpreter became available Internet searching via a mobile phone was also used. This was less used by elderly patients, so they were left more vulnerable Participants used control questions to determine if patients understood them Participants felt helpless at times if they could not properly communicate with patients even when an interpreter was present Both telephone and in‐person interpreters were used. More phone based during COVID‐19, which shortened the time between patient admission and the interpreter being available Phones created barriers as the interpreter was unable to view body language and would miss nuances that would be noticeable Can be a large difference in the technical competence of the interpreter Some participants mentioned that the interpreter is from a small community of people and knows the patient personally—patients would opt to reject an interpreter because they wanted confidential treatment Patient ethnicity: not discussed
Cioffi (2003)	Australia: peripheral hospital in Greater Western Sydney, general and midwifery clinical areas	To explore and describe nurses' experiences of communicating with culturally and linguistically diverse (CLD) patient population and their families	Design: Interpretive‐descriptive design	Purposive sampling. Participants: Registered Nurses (*n* = 11) Midwives (*n* = 12). Experience: 5–25 years (Md. = 10). Ethnic background: Anglo‐Centric (*n* = 13). Ethnic (*n* = 10): Asia, South America, Philippines, Sri Lanka, Europe, Indigenous Australian	Data collection: individual interviews (approx. 45 min). Data collection period: not reported. Data analysis: unitising and categorising	Preferred face to face and used telephone if not available especially in an emergency as found to be cumbersome Family, charts, sign and body language used as a strategy when HCI not available Limitations, time restraints on the interpreter service and the amount of info could be communicated effectively within the time limit Easier to access bilingual HCW's contributed to patient encounters communication by clarifying information at the point of care, identifying patient‐centred issues, improving patient care Bilingual nurses were also concerned on the quality of the information they were interpreting. Patients waiting for a bilingual nurse to start shift was of concern. Accessibility to get interpreters on weekends/night shifts, and knowing when they were required Issues of confidentiality and actual translation of what is being said Easy to access common languages Patient ethnicity: Chinese, Arabic, Vietnamese, Turkish, Bosnian only discussed
Coleman and Angosta (2016)	USA: 380 bed acute care hospital in western state	To explore the lived experiences of acute‐care bedside nurses caring for patients and their families with limited English proficiency	Design: qualitative exploratory using phenomenological approach	Purposive sampling. Participants: Registered Nurses (*n* = 40). Gender: Female (*n* = 39). Age (years): 25–62 (Md. = 43). Experience (years): 3–34 (Md. = 10). Ethnic background: Hispanic (5%), Non‐Hispanic (95%), Caucasian 80%, Asian 12.5%, more than one race 5%. Self‐reported primary language: English 87.5%, Tagalog 5%, Cebuano 2.5%, Chinese 2.5%, Korean 2.5%	Data collection: individual interviews (30–60 min). Data collection period: not reported. Data analysis: Van Manen's six‐step phenomenological method	Preferred face‐to‐face and most often used as added advantage of body language and facial expressions Telephones were cumbersome although convenient, helpful, necessary but not equal substitute for face‐to‐face Patient ethnicity: not reported
Fatahi et al. (2010)	Sweden: radiology departments in three largest public hospitals in Gothenburg	To explore nurse radiographer's experiences of examining patients who do not speak the native language (Swedish)	Design: qualitative	Purposive sampling. Participants: Nurse radiographers (*n* = 11). Age (years): 30–52 (Md. = 40). Experience (years): 2–30 (Md. = 13). Ethnic background: Non‐Swedish (*n* = 4)	Data collection: three focus groups (50–70 min). Data collection period: November 2007. Data analysis: qualitative content analysis	Preference was for professional interpreters, especially when complex procedures were being performed Interpreters should be trained in terminology and the principal ways of performing radiological examinations. Avoided children as too much responsibility for them In emergency situations family and friends were unavoidable Acknowledged the stress that using family and friends had on the family and friends when they needed to interpret Acknowledged the use of bilingual staff not ideal but often needed in an emergency situation Better quality communication when HCI used In the absence of an interpreter, body language was used to instruct the patient More complex examinations required more explanation Lack of time and interpreter unfamiliarity with radiological procedures were identified as problems Using an interpreter increased the time required for a procedure Patient ethnicity: Arabic only discussed
Feiring and Westdahl (2020)	Austria (Hospital A) and Norway (Hospital B). Both hospitals were large university hospitals in urban settings	To compare perceptions held by hospital managers and healthcare practitioners of the factors influencing the use of remote video interpretation and in‐person interpretation	Design: quasi‐experimental qualitative design. Purposively (non‐randomly) selected two hospitals located in Austria and Norway	Purposive sampling. Fifteen participants in total. Hospital A, *n* = 6; Hospital B, *n* = 9. Nurses (*n* = 4)	Data collection: 3 Focus groups. Guided by semi‐structured topic guides (one guide for managers and one for healthcare practitioners) that explored six main topics. (2 to 6 persons, 40 to 60 min) 4 individual interviews, 30 to 40 min. Data collection period: March and May 2018. Data analysis: predetermined categories based on the COM‐B and the Theoretical Domains Framework (TDF)	Ordering interpretation services was time consuming and created extra work Sometimes in‐person interpretation was favoured because video interpretation was perceived as too detached to the situation Cannot always get an interpreter on demand Positive experience—makes work easier. Reduced length of stay Video interpretation—it is a lot easier because we do not have to find somebody, call them and beg them to come in and help us. Emergency Department—Family the only alternative. Nurses relied on family to help interpret Patient ethnicity: not reported
Hemmila (2004)	USA: this study took place at an accredited, metropolitan. School of Nursing in a large Midwestern United States city. The school had two Registered Nurse (RN) Programs, a Bachelor of Science in Nursing (BSN) Completion Program and a Masters of Science in Nursing (MSN) Program	To describe the experiences of nurses who have worked with interpreters in health care	Design: exploratory descriptive (Questionnaire and one‐on‐one telephone interview)	Convenience Sampling: 40 BSN completion and MSN nursing students completed the survey. Ten subjects from the survey sample volunteered to participate in one‐to‐one in‐depth interviews. Age (years): 23‐57 (Md = *42*). Gender: 87.5% female. Ethnic background: 87.5% white, 12.5% African American. Years experience; Median 17 years. BSN completion: 37.5%. MSN Completion: 62.5%	Data collection: Questionnaire and telephone interview. Telephone interview lasted 30 min. Data collection period: not reported. Data analysis: grounded theory was used to analyse the interview data. A constant comparison method to analysis the transcriptions. Data were analysed using descriptive statistics	The findings revealed that nurses are aware of the importance of culture in providing care, but have varying levels of awareness about various cultures Overall, the results showed that nurses did not have a clear idea about the role of the interpreter The findings showed that nurses need ongoing cultural education despite having had some cultural training courses and in‐service classes Instruction should include not only how to work with an interpreter, but all aspects of the interpreter roles The findings suggested interpreters who worked for the hospital had a better relationship with nurses and were familiar with the procedures and the needs of the nurses, patients/clients, and families Nurses in this study found language lines helpful when limited time was a factor in providing safe care. However, handing the phone back and forth was awkward for the nurse and the patient/client especially when the patient/client was in a compromised condition ‘Live language’ services are preferred The findings of this study identified that family members and other health care personal are often used in the interpretive interview process. The findings revealed that nurses use families to obtain information, plan care, and receive education
Høye and Severinsson (2008)	Norway: 3 Intensive care units, 3 separate university hospitals	To explore nurses' perspective of their encounters with multicultural families in intensive care units in Norwegian Hospitals	Design: descriptive exploratory qualitative design with a retrospective focus	Purposive sampling. Participants: Registered Nurses (*n* = 16). Gender: Female (*n* = 15). Age: 25–64 years. Experience: 2–32 years (Md. = 7.6). Ethnic background: Norwegian	Data collection: Focus group interviews—two FGs met on three occasions and one FG met on two occasions (approx. 90 min each). Data collection period: not reported. Data analysis: interpretive content analysis	Forced to involve family as interpreters in daily caring Employees used as interpreters and the use of linguistic aids (use of tape recorder, pictures) Nurses concerned that the family did not understand the meaning of the information despite being fluent in Norwegian Patient ethnicity: Pakistan, Iraq, Vietnam, Somalia, Bosnia, Iran, Turkey, Serbia, Sri Lanka and Poland
Hsieh (2015)	USA: 784 bed academic medical centre in Oklahoma	To understand the variety of considerations and parameters that influence providers' decisions regarding interpreters	Design: qualitative	Sampling: not reported. Participants: Registered Nurses (*n* = 6). Gender, age, experience, ethnic background not reported separately for nurses	Data collection: eight focus groups and 14 individual interviews (60–90 min). Data collection period: not reported. Data analysis: grounded theory (constant comparative)	Delay in care was experienced when waiting for a professional interpreter causing frustration Would use bilingual staff members however concerned for co‐workers increased workloads and responsibilities Time constraints restricted use of interpreters Concerned about use of telephone as it poses a poor choice to meet patients' emotional needs Wouldn't use family for potentially life‐threatening issues and also because they would interfere with the patients' autonomy Patient ethnicity: Spanish and Hispanic only discussed
Keller and Carrascoza‐Bolanos (2023)	USA: Tertiary Care Hospital, California	Describe clinicians' perspectives about linguistic and cultural barriers to providing medication management, education, and counselling to LEP patients and caregivers	Design: qualitative	Participants: Registered Nurses (*n* = 3, 21%). Gender, age, experience not reported separately for nurses	Data collection: interviews via Tape‐a‐call phone recording app or Zoom (COVID). Lasted 60–90 min. Data collection period: March 2021–August 2021. Data analysis: Dedoose software to code and analyse the data. Constant comparison method	Perceptions of lack of time and urgent issues led to the use of family members by nurses Clinicians reported patient concerns regarding the trustworthiness of interpreters and healthcare professionals Clinicians expressed strong preferences for in‐person and virtual interpreter services over phone‐based services Clinicians noted specific communication challenges in patients with low health literacy Bilingual and multi‐lingual clinicians worried about the quality of interpretation used by formal interpreters Clinicians described inconsistencies in the translation of documents to the patients' and/or caregivers' preferred language
Lee et al. (2018)	USA, cardiovascular, general surgery and orthopaedic hospital wards	Assess effects of a bedside interpreter—phone intervention on hospital discharge preparedness among patients with limited English proficiency (LEP)	Design: mixed method (focus groups for staff)	Sampling: not reported. Participants: Registered Nurses (*n* = 14). Gender: Female (*n* = 13). Age, experience not reported. Ethnic background: 7 white, 6 Asian	Data collection: four focus groups (60 min). Data collection period: not reported (study completed 2012–2013). Data analysis: deductive approach	Phones convenient for communicating general everyday in‐patient communication Preferred in‐person interpretation for complex discharges, teaching and medications/interventions or if multiple family members present Preferred family for routine discharge, especially if family were involved in providing care Patient ethnicity: Chinese and Spanish
McCarthy et al. (2013)	Ireland: Health services in South‐West region	To describe nurses' experiences of language barriers and the use of interpreters	Design: qualitative descriptive approach	Sampling: purposive. Participants: Registered Nurses (*n* = 7). Gender: Female (*n* = 5). Age: not reported. Experience: 3–30 years. Ethnic background: Not reported	Data collection: individual interviews. Data collection period: not reported. Data analysis: thematic analysis	Interpreter may not be knowledgeable with health jargon and terminology Family and friends were used in an emergency, concern about information being withheld by family Interpreter allowed objectivity Lack of interpreter continuity Complex process for interpreter utilisation Patient ethnicity: not reported
Mottelson et al. (2018)	Denmark: University hospital in southern Denmark	To investigate the attitudes and experiences of the university hospitals charge nurses regarding the use of video interpretation for patient contact	Design: questionnaire with closed and open‐ended questions	Sampling: purposive. Participants: Charge Nurses (*n* = 78). Gender, age, experience, ethnic background: not reported	Data collection: electronic questionnaire with closed and 2 open‐ended questions. Data collection period: December 2014. Data analysis: thematic text analysis	Lack of training with equipment or how to communicate through an interpreter Patient state of health; nurses felt that critically ill or patients with cognitive disorders it was better to have face to face rather than video interpretation Too busy to use interpreters when behind in daily care. Easier to use family and other staff members Patient ethnicity: Russian, Chinese, Polish, Arabic, English, German and Spanish stated.
Nailon (2006)	USA: 4 ED departments in a northwestern state	To describe nursing care of Latinos in the emergency department to determine how care is planned relative to the patient's ethnicity, including linguistic abilities	Design: interpretive phenomenological study	Sampling: Not reported. Participants: Registered nurses (*n* = 15). Gender: Female 73%. Age: not reported. Experience: < 1–16 years (*M* = 6). Ethnic background: White and/or non‐Latino (67%), Hispanic and/or Latino (27%), American Indian (6%)	Data collection: group and individual Interviews; Participant observation. Data collection period: Not reported. Data analysis: thematic analysis	Nurses relied on interpreters to develop and maintain interpersonal connections Relied on interpreters to assist in forming accurate clinical impressions of patients Latino Registration staff were used as interpreters for limited hours to interpret for nurses and doctors Some nurses used their limited Spanish to interpret Nurses frustrated with being unable to verify accuracy of messages being relayed with healthcare interpreters Nurses preferred to use interpreters at discharge. Nurses perceived healthcare interpreters to not be engaged and lacking medical knowledge Consistency with the same interpreter enhanced the connection the nurse had established with the patient Believed hospital administrators frowned upon calling in interpreters because it would cost money Longer waiting times for Latino patients, proceeding to care without healthcare interpreters Nurses not sure when patients needed an interpreter Only face‐to‐face used Patient ethnicity: Latino only stated
Neiman (2019)	USA, Minneapolis: acute care setting (Excluded ICU and ED)	To explore the experiences of acute care nurses in providing basic palliative care to culturally diverse patients	Design: Qualitative, theory‐generating study. Hmong was chosen as it was the 2nd highest patient language spoken in 2 of the 3 hospitals	Sampling: Participants: Registered Nurses (*n* = 34). Female Gender (*n* = 32). Ethnic background: White (28), Black or African American (4), Asian (2). Experience (years): < 1 (2), 1–5 (10), 6–15 (14), 26–35 (1), > 35 (2)	Data Collection: Pilot focus group, 6 additional focus groups and 8 individual interviews. Data collection period: not reported. Data Analysis: thematic approach	Managing language barriers—Means to communicate—need to rely on communication devices as in person interpreters not available Communication devices ineffective, difficulty hearing leading to incomplete understanding and nurses lacked confidence that patients understood what was being said Interpreters when available were rushed, inadequate time to overcome language barriers Nurses drew pictures, used applications on their smart phones to communicate instead of using the organisations language line Limitations—predominately white female nurses Excluded ICU, ED
Suarez et al. (2021a) (Patient Education and Counselling)	USA, Minnesota:ICU	To understand the perspectives of physicians, nurses and interpreters about the professional interpreters' role at end of life and critical illness discussions with patients and families who have LEP: to explore physicians, nurses and interpreters' perceptions about the advantages and disadvantages about the diverse interpretation modalities	Design: Grounded Theory. Secondary analysis	Sampling: Purposive. Participants: Registered Nurses (*n =* 12). Female Gender: 92%. Age: 20–35 years. Ethnicity: Not reported	Data Collection: individual, semi‐structured interviews (30 min). Data collection period: November 2017–April 2018. Data analysis: open axial and selective coding using NVivo (Version 11)	Different interpretation modalities influence communication dynamics with patients and families in the ICU Nurses perceived interpreters as a cultural broker and trust builders Nurses preferred in person interpreters to video conferencing interpretation as this promoted family and cultural understanding and interpreters were seen as a familiar face Nurses perceived video conferencing interpretation (iPads) as convenient due to its availability (24/7), but did not see this as a substitute for in person interpreters Nurses preferred a family member to be present during interpretation to ensure nuances were not lost in the translation
Suarez et al. (2021b) (*Journal of Critical Care*)	USA: Three ICUs, Mayo Clinic Rochester, MN	To assess clinicians' and interpreters' perspectives of suboptimal communication and their recommendations to improve communication between the healthcare team and patients and families with LEP in the ICU	Design: Qualitative	Sampling: Purposive. Participants: Registered Nurses (*n =* 12). Female: 92%. Age: 20–35 years. Ethnicity: not reported (US born)	Data collection: Individual, in‐person, semi‐structured. Data collection period: November 2017–April 2018. Data analysis: open axial and selective coding using NVivo (Version 11)	Suboptimal assessment and treatment of patient symptoms (‘I felt bad that patient went two and a half hours feeling like her environment wasn't comfortable for her. I truly just did not know. I didn't realise there were specific ways she wanted her environment’.) Unmet patient and family expectations Decreased patient autonomy Unmet end of life wishes Clinician distress (‘It can be hard to hold your own feelings in check when you're (like) torturing this person’.)
Watts et al. (2018)	Australia: three teaching hospitals	To explore organisational and systemic challenges encountered by health professionals working with patients from ethnic minorities	Design: Grounded theory	Sampling: purposive. Participants: Registered Nurses (*n* = 21). Gender, age, experience, ethnic background not reported	Data collection: individual interviews (20–60 min) and focus groups (30–90 min). Data collection period: May–October 2013. Data analysis: grounded theory constant comparative method	Telephone interpreters were inadequate for detailed discussion; lack of verbal cues Use family when interpreter unavailable however concerned about accuracy, understanding and information being withheld Using interpreters required more time Okay to use telephone for basic side effects and getting questions answered Spent more time with interpreters to build rapport Privacy issues with rare dialect interpreters and information being shared within their social group Patient ethnicity: NR

Nine studies were undertaken in the United States (US), four studies in Scandinavia (Denmark, Norway, Sweden), two in Australia, one in England, one in Ireland, and one in Austria. Although the number of languages spoken by patients was not always reported, the US studies primarily discussed the need for Spanish‐speaking interpreters with patients being either Latino or Hispanic, or Chinese interpreters with patients speaking either Mandarin or Cantonese. One US study was undertaken among Hmong patients.

The three Scandinavian studies reported more diverse language groups with people speaking Arabic, Serbo‐Croatian, Kurdish, Persian (Farsi), Somali, Russian, Chinese, Polish, English, German and Spanish. Studies also referred to patients from the following countries: Pakistan, Iraq, Vietnam, Bosnia and Herzegovina, Iran, Turkey and Sri Lanka.

In one of the Australian studies, nurses discussed the need for healthcare interpreters (HCIs) for diverse language groups including Arabic, Bosnian, Chinese (Mandarin, Cantonese), Slovakian, Somali, Turkish and Vietnamese. The study from England discussed the need for African, West Indian, Indian, Arabic and Chinese‐speaking interpreters. The study from Ireland did not specifically state the most common language group; however, it noted an increase in immigration and referred to a Bosnian‐speaking interpreter in the findings of the study.

Ten studies included only nurses in the study sample, while the eight remaining studies contained mixed samples of nurses and other health professionals (primarily physicians), where nurses' responses were clearly identifiable. The most common methodology was qualitative descriptive, with six studies collecting data by individual interview, seven by focus groups, three using both individual interviews and focus groups, one using mixed methods, and one using a questionnaire with two open‐ended questions.

Four themes and associated sub‐themes were identified, which are described in the following section with illustrative quotations.

## 4 Theme 1: Working With Interpreters—‘An Impartial Bridge’

Nurses recognised that healthcare interpreters (HCIs) offered valuable and useful information, particularly in complex situations, providing a more comprehensive understanding. However, for minor interactions, nurses chose to manage without an interpreter. The success and satisfaction of interpretation encounters were enhanced when the same interpreters were used consistently for patients and when interpreters were familiar with the clinical setting and context.

### 4.1 Subtheme 1: Facilitating Communication—‘They Help Me Do My Job’

In all studies included in this review, nurses acknowledged the usefulness, positive attributes and valued the support provided by HCIs which helped them to do their job. Hsieh (2015), Lee et al. (2018) and Fatahi et al. (2010) discussed clinical complexity and found that HCIs were an ‘impartial bridge’ to the communication of assessments, medication, life‐threatening management and discharge treatment. Nurses in Hemmila's (2004) study reported they were able to give better care and enhance the quality of care because they had ‘a more complete picture’ of what their patients' needs were.

Fatahi et al. (2010), Hsieh (2015) McCarthy et al. (2013), Nailon (2006) and Cioffi (2003) identified that HCIs provided accurate translation to ensure understanding of the procedure about to occur. For example, Fatahi et al. (2010) reported nurses believed a professional interpreter was essential to inform patients about procedures, particularly in relation to side effects and complications. Similarly, nurses in Feiring and Westdahl's (2020) study reported using interpreters avoided misunderstanding and made their work easier.

Despite their usefulness in helping nurse ‘do their job’ nurses were conscious of their use in rare dialects and small community groups. Nurses reported patients' concerns about confidentiality and privacy issues (Ali and Watson 2018) and ‘information getting back to the community’ (Cioffi 2003; Watts et al. 2018).

### 4.2 Subtheme 2: Decision Making—‘I'm Not Going to Call an Interpreter Up Just for Two Sentences’

Nurses made clinical decisions on when a HCI would be required based on the complexity of the procedures being undertaken. Hsieh (2015) highlighted how clinicians engaged in ‘calculated use of professional interpreters’, including assessment of clinical complexity. Tasks considered minor for example a couple of sentences asking a patient to roll onto their back did not require an interpreter. In contrast, more complex tasks such as life‐altering procedures would require a professional interpreter. Similarly, Fatahi et al. (2010) reported radiology nurses preferred professionally trained interpreters when complex procedures were undertaken, such as radiological examinations using contrast medium. Nurses preferred to use professional interpreters to explain ‘normal side effects’ versus potentially life‐threatening adverse reactions. As one nurse commented ‘If you don't know what this is, you may think that you are going to die or something like that…and you may then try to get up from the examination table and thereby ruin the examination’ (Fatahi et al. 2010). Nailon (2006) described how nurses also decide whether to use a HCI based on what could be complex discussion, and to help inform accurate clinical impressions of patients.

Nurses identified using the same interpreters for patients and/or interpreters being familiar with the clinical setting and context increased the success of and satisfaction with interpretation encounters. Nailon (2006) reported nurses found that ‘consistency with the same interpreter enhanced the connection the nurse had established with the patients by affording the interpreter familiarity with the patient's situation’. Nurses noted an additional benefit was interpreter consistency and familiarity increased patient comfort in unfamiliar environments (Hemmila 2004).

## 5 Theme 2: Tensions and Challenges—‘Did I Just Miss an Important Piece of Information?’

Although nurses had positive experiences working with HCIs, they also experienced frustrations. This included the accuracy of interpretation, an understanding of medical and nursing terminology, feeling sidelined, and a lack of technology and equipment to help the interpretation process. Nurses who had less experience working with HCIs felt left out of the encounter. The process of booking an HCI and the time they were available was another source of frustration for the nurses. The booking and use of HCIs were influenced by time.

### 5.1 Subtheme 1: Unfavourable Encounters—“It's Frustrating”

A number of studies reported examples of nurses who experienced frustration when using interpreters. In a phenomenological study exploring nurses' experiences of caring for Latino patients in an emergency department (ED), Nailon (2006), found nurses often felt frustrated with interpreter accuracy which at times impacted on the delivery of nursing care. One nurse commented ‘I feel very frustrated sometimes when I'm not sure they [interpreters] understand what we are saying’.

Nurses reported feeling ‘sidelined’ at times during the information exchange: ‘They'll be interpreting for me, and then all of a sudden they have this little interchange without me being involved at all. And I'm like Okay, wait a minute. Back up. Did I just miss an important piece of information?’ (Nailon 2006). Interestingly, in this study nurses who were less experienced in working with interpreters were more likely to feel left out of encounters.

Professional interpreters who had an inadequate understanding of medical and nursing terminology were a source of frustration for nurses. Nurses reported working with interpreters who misinterpreted information that was being conveyed to patients which resulted in miscommunication and ultimately safety and quality of patient care. For example, one nurse described how ‘a patient was booked for a cystoscopy but her interpreter told her that she was going for a gastroscopy. The patient thought that they were putting the camera from her mouth to stomach, but of course this was not the case’ (Ali and Watson 2018).

Nurses were also likely to report less positive experiences if interpreters stood out of view of patients, seeing this as disrespectful and likely to convey the impression of being disengaged in the encounter. One nurse commented ‘I know it won't make me feel like, you know, if I went somewhere and people were talking to me through a curtain or a wall or whatever, that it really mattered what I was saying anyway cause they weren't really paying attention. That could give you a bad feeling’ (Nailon 2006).

Finally, the use of technology for interpretation was also described as frustrating due to the lack of accessibility of three‐way phones and the video equipment breaking down. Nurses in Mottelson et al.'s (2018) study reported they did not have access to a help desk and there were also issues with poor internet connection and ‘because of this we don't have access to the internet in all rooms. Therefore we only have one room for video equipment in the entire department’. Charge nurses emphasised reasons for not using video interpretation included staff feeling insecure and lacking skill, summed up in the following quote: ‘We are facing technical problems—we are not trained to deal with such issues’.

### 5.2 Subtheme 2: Lack of Availability—‘On‐Call Basis Only’

Nurses described access to interpreters only available on an on‐call basis as a significant barrier to their use. One nurse commented that hospital administrators frowned upon nurses calling in interpreters if they had in‐house bilingual staff: ‘but they [administrators] would be like, “Well, you just gotta get by, or call somebody from medical” [other unit in hospital]. Cause it would cost them money to have the interpreters come in’ (Nailon 2006).

Accessing professional interpreters was further complicated when external agencies arranged interpreters on a sessional basis: ‘Access and utilization of formal interpreter services is far from simple. The organization of interpreters was managed through an agency on a sessional basis’ (McCarthy et al. 2013).

Lack of availability of interpreters out of hours, particularly during night shifts and on weekends, was highlighted by nurses as a barrier to using professional interpreters (Ali and Watson 2018), and this was compounded by not being able to predict when an interpreter would be required (Cioffi 2003). In language groups where few interpreters were available, nurses reported patients not accessing interpreters during their hospital admission, or if they did, it was not until the day of discharge (Cioffi 2003).

### 5.3 Subtheme 3: The Impact of Time—‘Time‐Bound’

Even when professional interpreters were available, the time taken to arrange for a professional interpreter and the limited time that nurses could use them were identified as barriers. In the study by Hsieh (2015) one nurse commented ‘I would use [professional interpreter] SO MUCH MORE OFTEN if they were just right there where I could just say, “Hey, can you come here? I need to ask you something real quick” instead of having to call and wait 25 min for them to get here’. Similarly, Ali and Watson (2018) identified the limited time nurses could use professional interpreters as a barrier noting the service was ‘time‐bound’ and could only be booked for an hour thus potentially impacting on care. Fatahi et al. (2010) and Neiman (2019) also identified barriers relating to using professional interpreters when the interpreters were time‐constrained, particularly if they were not able to arrive at the beginning of the consultation. The lack of time available with interpreters meant the consultations were rushed, which didn't allow the necessary time to understand what patients were experiencing (Neiman 2019). Nurses would also attempt to maximise the time they had by extracting all information: ‘Eventually we got an interpreter in… and we asked every question that we possibly could… we just focused and jumped in with all the questions’ (McCarthy et al. 2013).

The time taken to book and the wait for an interpreter were identified as barriers to using interpreters. In several studies, nurses noted ‘the effort to get an interpreter’ (Feiring and Westdahl 2020), particularly in acute situations. In a study of ED nurses, a lack of readily available interpreters posed particular difficulties for nurses when caring for patients admitted to the ED with traumatic injuries or high acuity presentations. These situations required nurses to be vigilant and remain with their patient however, they needed to leave their patient to seek out an interpreter: ‘You're sort of saying, “Just a minute, I know you want to tell me something. I will be back.” And they don't know what you're saying. You can't even say, “You're important to me. Let me find an interpreter so we can talk.” It happens all the time’ (Nailon 2006).

## 6 Theme 3: Making Connections—‘A Personal Relationship’

Communicating with interpreters can take several modes, including face‐to‐face, telephone or video interpretation. Ultimately, choosing the appropriate mode is dependent on the situation; however, clinicians were generally limited in their options, which impacted their communication.

### 6.1 Sub‐Theme 1: More Than Words Can Say—‘We Can Look at the Face’

Nurses overwhelmingly preferred health care interpreters to be present for face‐to‐face interpretation. This was particularly important if patients were critically ill (Mottelson et al. 2018), cognitively or hearing impaired (Ali and Watson 2018; Mottelson et al. 2018), required complex discharge teaching (Lee et al. 2018) or were at end of life (Suarez et al. 2021b). For example, Lee (2018, 29) described nurses' preferences for face‐to‐face interpreters particularly when more than one family member was involved in discharge preparation, ‘…it is so much easier to have someone in person because otherwise you have people asking questions, and the interpreter (over the phone speaker) doesn't even know who they are talking to, because there are three or four different voices…then people are like‐all sort of talking and they just interrupt’. Coleman and Angosta (2016) also reported nurses' preferences for face‐to‐face interpreters; ‘you can see if they have that kind of quizzical look on their faces when they are talking about something and trying to process it. I mean, you get all of those cues back’.

In the study by Mottelson et al. (2018) charge nurses were satisfied with the use of video interpretation which ‘helps with [maintaining] a good dialogue between doctor, patient and nurse’. However, more than two thirds of charge nurses still preferred to use face‐to‐face interpreters for critically ill patients or when there was complicated information which needed to be explained.

In the study by Hsieh (2015) that explored provider's decision making about interpreter use, nurses indicated a preference for on‐site (face‐to‐face) interpreters over telephone interpreters. One nurse commented: ‘Well, there's just a personal relationship built up with our translators. We know each other. We've learned each other's styles of communicating cause we all do it differently. Some of us are very, you know, step by step and some of us are very casual. So we build up a knowledge of how we communicate’ (Hsieh 2020). Similar preferences were reported by nurses in Hemmila's (2004) study who preferred face‐to‐face interpretation over telephone interpretation which they described as ‘impersonal because you can't see that person’.

Nurses in Hsieh's (2015) study also preferred face‐to‐face (on‐site) interpreters because this allowed patients to ask further questions ‘You know, I'd say [to the telephone interpreter], “You're sure that she doesn't have any more questions?” They said, “no.” But when somebody is on‐site, one thing I have noticed is that they have a tendency to maybe ask one or two more questions. They do, versus being on the telephone’ (Hsieh 2020). Suarez et al. (2021b) noted similar concerns by nurses who described the negative effects of remote interpretation which didn't ‘help with the cultural context of things’. Nurses in Feiring and Westdahl's (2020) study also favoured in‐person interpretation with video interpretation perceived as too detached—‘you lose personal contact’, and telephone interpretation used only when there was an emergency.

### 6.2 Subtheme 2: Nuances of Telephone Interpretation—‘It Is a Three‐Way Conversation’

Seven studies reported nurses' experiences of and preferences for using healthcare interpreters via telephone. Generally, nurses viewed telephone interpreters as useful particularly for situations that required factual information, such as pain management and decision making (Lee et al. 2018) and reconfirming information previously discussed using a face‐to‐face interpreter (Ali and Watson 2018; Watts et al. 2018). For example, in the study by Watts et al. (2018), the majority of nurses felt that due to the inability to exchange non‐verbal cues, telephones were inadequate for more detailed or sensitive discussions: ‘it (Telephone interpreting services [TIS]) just wouldn't work, but for those subsequent follow up appointments when you're asking the basic side effects in getting questions answered you know back from the patient, yeah they seem to work okay’.

However, Cioffi (2003), Hemmila (2004), and Coleman and Angosta (2016) also described nurses' experiences of using phone interpreters to be awkward and time consuming. A nurse in Coleman and Angosta's (2016) study explained that although the phones worked well ‘it's just cumbersome—it's, you know, hard to hold the phone and find the phone and I think it's intimidating to patients to talk to a stranger on the phone about these personal issues too that they're not really sure that they're speaking to somebody on the phone’. Lee et al. (2018) also reported nurses found using telephone interpreters tedious: ‘it's a three‐way conversation. And I find it's much easier if there is a person on‐site. And so, we can look at the face, and we can interpret and say, “Did they really understood [sic] what we say?”’.

Cioffi (2003) and Hemmila (2004) also reported nurses were more likely to use a telephone interpreter in an emergency situation however, there was concern that using telephone interpreters posed a poor choice to meet patient's emotional needs and it was more difficult to develop trust. One nurse stated ‘on site interpreters make the patient feel better as opposed to [telephone interpreter who are] just are more factual’ (Hsieh 2015). Nurses in Hemmila's (2004) study reported ‘the trust develops much easier in person than it would on the telephone’.

## 7 Theme 4: Workarounds—‘We Just Had to Deal With It’

Whilst nurses valued the support provided by HCI, issues related to availability and the need for timely access meant they often needed to find workarounds to ensure effective communication with patients. Commonly used strategies comprised using family and bilingual colleagues to bridge the language gap.

### 7.1 Subtheme 1: Using Family and Friends—‘It Happens—We Use the Family’

Thirteen of the 18 studies in this review reported using family and friends was unavoidable when an interpreter was not available, and nurses ‘just had to deal with it’ (Keller and Carrascoza‐Bolanos 2023). In some settings, for example the ED, family were used in the first instance before requesting an interpreter: ‘If they didn't have a relative or if you felt that…you weren't getting the right information…you would get an interpreter’ (McCarthy et al. 2013).

Family members were preferred when providing daily care and were considered ‘quite handy for communicating everyday things’ (Cioffi 2003). In the study by Mottelson et al. (2018), charge nurses reported that it was easier to use family members because they were already present. This was particularly the case when situations were acute and there were difficulties in booking an interpreter quickly. In the same study, nurses preferred to use family members in daily tasks ‘as it was easier and saved time to use the patient's relatives or friends’.

Nurses reported that some patients wanted to use their family to interpret. In the study by Hsieh (2015), the author reported in the Hispanic culture male family members are often the decision makers for female patients ‘providing a comforting and reassuring presence’ that facilitated patient receptiveness. Some nurses however preferred not to use family interpreters as they felt this compromised patient autonomy: ‘The family gentleman was there, and I had to talk through him, and that's very annoying. But [the patient] didn't want me advocating for her’.

Nurses also reported that family members' ability to provide emotional support and security to the patient was taken into account when deciding to use family members instead of interpreters (Fatahi et al. 2010; Hsieh 2015). Family members were also often preferred during routine discharge, especially if they were going to provide care for their relative at home (Lee et al. 2018).

In a study of 16 intensive care unit nurses (Høye and Severinsson 2008), nurses relied heavily on the input of family and friends. Participants reported because they were at the bedside 24/7 it was impractical to use interpreters to provide all information, thus they were ‘forced to involve the family member as interpreters in daily caring’. This was despite being uncertain that family members used as interpreters may not have actually understood the information being conveyed; ‘our experience is that families who seem to speak fluently don't catch the differences in our language when we talk to the patient’ (Høye and Severinsson 2008). Similar finding were reported by Suarez et al. (2021b) in a qualitative study of the health care team's perceptions of the role of interpreters during end of life and critical illness discussions in the intensive care unit. Nurses in this study preferred not to use family members stating ‘I don't really feel like that is a great thing to use family as interpreters…I don't know what they are saying, and if they're truly telling what I am saying’.

Although nurses often preferred using family members—and family members themselves encouraged the nurses to ‘call me at any time’ (Keller and Carrascoza‐Bolanos 2023), they also acknowledged the dilemma this created particularly where family or friends censored or withheld information, became emotionally involved or were unable to remain neutral. Nurses also reported concerns about the ability of family and friends to understand the information being communicated and when they believed they ‘weren't receiving the right information’ (McCarthy et al. 2013) nurses would call an interpreter.

Nurses acknowledged that using family and friends could cause stress for the family/friend which in turn could be conveyed to the patient. However only one study identified nurses' concerns about using children as interpreters, with one nurse participant commenting ‘I never want to see a child as interpreter; it is a terrible moment for me’ and ‘Perhaps they do not interpret all the way, if they feel it does not sound good for Mummy’ (Fatahi et al. 2010).

Nurses made calculated decisions when deciding to use a family member to interpret: ‘it is one thing to use a family member to see if they like to crochet, but another to ask if they are allergic to something, which could potentially be a life‐threatening issue’ (Hsieh 2015). However, nurses recognised that procedures involving informed consent always required interpreters and wherever accurate information exchange was needed stating ‘I make sure I have an [professional] interpreter and make sure [the patient] understood everything’ (Hsieh 2015).

### 7.2 Subtheme 2: Using Bilingual Staff—‘They're Sort of a Go‐Between’

Four of the studies reported nurses using in‐house bilingual staff, despite not knowing whether these staff received any formal training. For some nurses, this caused concern with uncertainty about the accuracy of interpretation. One nurse commented: ‘…you kind of lose it with some of the specific body parts…pretty soon you just get stomach instead of appendix or gallbladder or liver…’ (Nailon 2006). Concerns about accuracy of interpretation were also expressed by nurses in Hemmila's study who queried documenting ‘second hand information’ if they were ‘unsure of the accuracy’ (Hemmila 2004).

In the study by Cioffi (2003), bilingual healthcare workers (HCWs) based in the study setting (peripheral hospital) were used by nurses when HCIs were not available. Nurses reported that using bilingual HCWs resulted in the delivery of patient centred care that was culturally congruent. Nurses found bilingual HCWs particularly helpful in complex and emotional situations. One nurse stated: ‘they're sort of a go‐between the family and what is perceived institutional. It just diffuses the situation’ (Cioffi 2003). In addition to using bilingual HCWs, the nurses in Cioffi's study also discussed the use of bilingual nurse colleagues as interpreters. Nurses described the benefit of using bilingual colleagues in reducing anxiety, yet this strategy had consequences such as patients waiting for specific bilingual nurses to return to work to communicate concern. Both bilingual and non‐bilingual nurses expressed concern at the risk of delaying communication.

An unintended consequence of using bilingual HCWs as interpreters is the impact on workload. Fatahi et al. (2010) reported the use of bilingual HCWs caused increased stress due to the time required to interpret and the interruption of workflow, as it was not part of routine work. Nurses were also concerned about asking their bilingual colleagues to interpret as they didn't want to increase their colleague's workloads and responsibilities and would only ask bilingual colleagues to interpret ‘a couple of things’ as opposed to something like discharge instructions because they would ‘take(s) about 5–10 minutes’ (Hsieh 2015).

## 8 Discussion

This review of qualitative evidence was undertaken to better understand nurses' experiences of and preferences for using healthcare interpreters when communicating with patients with limited proficiency in the primary language in acute care settings. Eighteen research papers from eight countries were included in the review, representing nurses from a broad range of specialties including medical‐surgical, oncology, critical care (ICU, ED, PACU), cardiology, maternal‐child, radiology, and mental health. Overall, the included studies met the CASP criteria; however, half (*n* = 9) failed to provide sufficient detail regarding the relationship between the researcher and participants.

Overall, the review highlighted that nurses experience a range of challenges in accessing and working with healthcare interpreters. These challenges could be clustered as those related to the healthcare system, time required, and information overload; those related to the healthcare interpreters; and those related to the individual practices and preferences of nurses.

### 8.1 Healthcare System

At a system level, nurses reported significant challenges in accessing healthcare interpreters due to time‐consuming processes and lack of availability, and for many, this resulted in them using family members for acute situations where immediate communication with their patient was essential. Most healthcare organisations' interpreter policies discourage the use of family, friends, or unqualified interpreters, citing their use places a significant burden on the person interpreting (who may be exposed to sensitive or inappropriate information), information transfer cannot be guaranteed, and it changes the dynamics and power balance of the consultation (Queensland Health Interpreter Service 2007).

Interestingly, few studies have demonstrated adverse outcomes when family members were used to interpret. Flores et al. (2012) used a cross‐sectional error analysis of audiotaped emergency setting visits to show the proportion of errors of potential consequence was significantly lower for professional (12%) versus ad hoc (family members and bilingual staff—22%). In a study of audiotaped consultations with immigrants diagnosed with incurable cancer, Butow et al. (2011) reported 10% of consultations involving family members or health professionals could have resulted in misunderstanding. Nevertheless, the authors still concluded that there was likely a role for family and there is an increasing body of literature arguing for the use of family members as interpreters, suggesting that in some contexts, this may be preferable (Ho 2008). When professionals deny family involvement in the name of protecting patients' safety, autonomy, and confidentiality, they may ironically be exacerbating their vulnerability and perpetuating their marginalised status (Ho 2008). The arguments here are not to suggest that an informal interpreter is suitable for all interpreter‐mediated health and social care encounters, but that individuals have the legal right to make that choice, whether professionals agree or not, and that where there are concerns, existing statutory duties enable inquiries to take place (Pollock 2020).

Policies are beginning to reflect patients' right to choose whether they wish to use a HCI, bilingual health professional or a family member when interpretation is required. In these circumstances it is recommended that staff clearly document in patient's progress notes that a professionally trained interpreter was not available (NHS England/Primary Care Commissioning 2018; Queensland Health Interpreter Service 2007). In some countries, health systems are responding to the increasing linguistic diversity of the population by training and certifying clinicians in medical interpretation, referred to as dual‐role staff‐interpreters (Moreno et al. 2007; Villanueva 2023). While these programs are currently institution‐specific, contributing to a lack of quality assurance and variability in levels of competence, it is possible that with standardised training and testing, the skills of bilingual staff may be used to improve the quality of patient care and satisfaction (Moreno et al. 2007).

### 8.2 Time Required and Information Overload

For nurses who were able to access a HCI, the limited time available meant they maximised the time they had by asking every possible question. In a study of women's experiences of receiving maternal care, nurses reported women were often overwhelmed by the number of clinicians who took advantage of having an interpreter present to ‘pack as much information into one consultation as possible’ (Origlia Ikhilor et al. 2019). Guidelines suggest additional time is needed when working with interpreters, typically double that required if an interpreter is not needed (NHS England/Primary Care Commissioning 2018). However, in practice this is rarely taken into account when allocating patient loads in acute care settings. In the study by Nailon (2006), nurses verbalised concerns about delayed care and reduced time spent at the patient's bedside when interpreters were not available, a finding supported in a scoping review by Gerchow et al. (2020) which reported nurses modifying care delivery and spending less time with non‐language concordant patients. The additional time required to care for patients when interpreters are not available can also reduce efficiency of hospital systems, highlighted in a study by Clayton et al. (2016) where perioperative nurses reported the need to temporarily close recovery when all staff were occupied in managing a postoperative patient from a different language background.

Although this review did not explore the experiences of patients when engaging with HCIs, patients have described similar experiences to nurses when time with interpreters was limited, including inaccurate translation (Brooks et al. 2016) and feeling rushed—needing essentially double time because it requires ‘double effort’ (Sturman et al. 2018). From the interpreter's perspective, limited time with patients has been shown to result in information overload (Abbe et al. 2006) and increase physical, emotional, and mental stress (Yick and Daines 2019).

A review by Khaleel et al. (2020) found that information overload negatively impacted the patient's decision‐making ability, psychological wellbeing, and comprehension of health information. For this reason, nurses need to consider prioritising what has the most immediate impact when working with interpreters and organising this to reduce information overload for both the patient and the interpreter (Kamara et al. 2018). Health care organisations should also consider the additional time required when caring for patients with LEP and reduce nurse‐patient ratios to ensure sufficient time to meet patient needs.

### 8.3 Healthcare Interpreters

Nurses' experiences of working with HCIs was overwhelmingly positive—they found them useful and valued the support they provided which helped them to ‘do their job’. They were particularly valued in situations where communicating information about complex procedures was critical, for example, in EDs and when interventional procedures were required. Three studies in this review reported nurses' preferences for using healthcare interpreters when complex discharge teaching was required. Of note, none of the studies in this review reported nurses' experiences of utilising healthcare interpreters when conducting an admission history for LEP patients admitted to the ward. Given research demonstrates the use of an interpreter on admission reduces the risk of readmission and can shorten the length of stay compared to patients who do not receive an interpreter (Lindholm et al. 2012), health services should consider models of care in which nurses are required to use interpreters when admitting patients.

Although only one study in this review of nurses' experiences of working with interpreters in acute care facilities reported an account of feeling sidelined when working with interpreters, studies undertaken in other settings and involving non nurses have included reports of sidelining during patient‐interpreter interactions. In a study of a home visiting program for young first‐time mothers Barnes et al. (2011), reported nurses' experiences were not always positive, with three of the 17 family nurse practitioners interviewed experiencing being sidelined. This resulted in one nurse deliberately arriving at the house early to have time alone with their client. Similarly, Hudelson et al. (2013) reported interpreters having side conversations with patients, which resulted in the clinicians' (doctors) being excluded from the conversation.

It may be that the interpreter does not understand what they have been asked to interpret and are trying to explain it. However, if HCIs go off on a tangent they may miss important information. Sidelining may also result in nurses being less likely to use HCIs in the future suggesting training nurses to explain the context and ‘set the scene’ for the interpreting encounter may help reduce this negative experience.

Patients who share a common language with interpreters may feel comfortable enough to engage in side conversations; however, it is also possible that clinicians less skilled in working with interpreters will experience sidelining. This suggests a need for clinicians to undertake training on how to work with interpreters, but equally, a need for interpreters to be aware of the negative impact on the nurse–patient interaction when they engage in side conversations with patients.

### 8.4 Individual Practices of Nurses

Perhaps the most interesting finding from this review was that nurses accepted it was inevitable that family would be used to interpret. Nurses made what Hsieh (2015) describes as ‘calculated decisions’ when deciding to use a professional interpreter. Similar decision‐making processes have been reported where nurses described measures ranging from the use of gestures to attend to patients' basic needs such as hunger and hygiene to using other patients for simple matters but not using these methods for complicated situations (the inference being that HCIs would be used) (Plaza del Pino et al. 2013).

For many, time constraints and what was perceived as less complex care resulted in nurses choosing a family member as their first preference for interpreting. There was also evidence in this review that nurses were using relatives to interpret because in some groups this was supporting cultural norms (Hsieh 2015), or nurses perceived family members as providing cultural and emotional support (Suarez et al. 2021a). This may be particularly important in some communities where it is a cultural norm for family members to take care of their parents, and this extends to interpreting (Hilder et al. 2019), particularly given family members have been shown to provide a sense of security that can only be supplied by those nearest to them (Fatahi et al. 2010).

The preference to use family members appears not to be restricted to nurses, with medical and allied health clinicians reporting the use of family members and friends in not only providing interpretation but also providing compassion and empathy (Hsieh 2015) and patients themselves also indicating a preference for family members to interpret, particularly where knowledge and trust were important (Hilder et al. 2019).

Although nurses frequently use family members as interpreters in health care settings, empirical data on the outcomes for patients when a family member is used versus an HCI is limited. In a quality improvement study reported by Wasserman et al. (2014) the authors identified the use of family members or friends as a common cause of errors or potential errors for LEP patients. The use of professional interpreters, when compared to ad hoc (family members or friends) and no interpreters, significantly reduced the likelihood of errors of potential clinical consequence (Flores et al. 2012). While family members can make valuable contributions by providing additional information (Meyer et al. 2010), families can impose their own agenda (Leanza et al. 2010) and may not accurately interpret the encounter and respond without confirming with the patient (Flores et al. 2012). The use of family members is a double‐edged sword, both increasing the risk of interpretation errors and reducing this risk by providing additional information or clarification. Acknowledging this dilemma, some organisations have responded by providing guidelines for when unaccredited bilingual speakers may be used, for when accredited HCIs are not available. Health professionals may be used for simple day‐to‐day communications followed by relatives or friends (NHS England/Primary Care Commissioning 2018; Queensland Health Interpreter Service 2007).

This review identified that nurses overwhelmingly preferred face‐to‐face interpreters to telephone interpreters, as nurses were able to observe nonverbal cues which may indicate confusion, for example, quizzical looks. Face‐to‐face interpreters were also preferred as nurses felt they were more likely able to meet the patients' emotional needs. This was particularly important when delivering sensitive or distressing news as it was not possible to observe patients' body language or try to interpret their reactions. While technology has enhanced nurses' ability to communicate with patients who have LEP, nevertheless, the lack of personal closeness and difficulty establishing rapport needs to be taken into consideration when alternate approaches to face‐to‐face interpretation are used (Hsieh 2015).

Healthcare interpreters themselves have also reported preferring face‐to‐face encounters over telephone interpretation, as it allowed for observation of nonverbal cues and reduced stress (Butow et al. 2012). Patients have also reported preferring face‐to‐face interpreters compared with telephone interpreters. In a systematic review of patients' satisfaction with telephone or video interpreter services compared to in‐person services, the authors reported higher satisfaction with face‐to‐face interpreters compared with telephone interpreters (Joseph et al. 2018).

The recent COVID pandemic has highlighted the need to deliver care virtually, resulting in an increase in the use of technology‐supported health care interpreter services (Westmead Redevelopment 2020). Some healthcare organisations have seen a three‐fold increase in video interpreting (South Western Sydney Local Health District 2020) and have begun developing guidelines for when face‐to‐face interpreting should occur and when it is clinically appropriate for video or telephone interpretation, suggesting that the interpretation landscape is likely to further change.

While three of the studies in this review identified nurses' preference for interpreters consistently allocated to a clinical area to increase their familiarity with medical terminology, it was also clear that familiarity could ‘breed contempt’ with interpreters providing additional information to what was relayed by the nurse (Hsieh 2007). Hsieh (2007) has also reported interpreters editorialising and volunteering information when interpreting for clinicians, which could have adverse clinical consequences.

Finally, this review highlighted the use of bilingual HCWs was a first preference for many nurses as they were more convenient to access than HCI and useful when performing simple or non‐complex procedures such as assessing pain control, comfort and performing activities of daily living. Two studies undertaken in the United States (Hsieh 2015; Nailon 2006) reported financial limitations related to the employment and use of professional interpreters influenced nurses' decisions to use bilingual HCWs. This finding is supported by a clinician survey investigating the use of interpreters at a 2000 bed public hospital in Switzerland where respondents indicated they were expected to ‘exhaust other strategies before calling a professional interpreter due to budgetary constraints’ (Bischoff and Hudelson 2009).

In addition to budgetary constraints bilingual nurses were also used to interpret because of their medical experience and knowledge of diagnoses and procedures (Hsieh 2015), they were also used as first choice when there was clinical or task urgency or hospital workflow pressures (Chang et al. 2019). This was despite nurses concerns they were breaching policy but their desire to support their colleagues to provide timely culturally competent care overrode these concerns (Chang et al. 2019). This study also highlighted a number of flawed assumptions held by clinicians, including that bilingual nurses were sufficiently fluent in the required language to interpret medical terminology. An additional concern highlighted in Chang et al.'s (2019) study was that bilingual nurses had the communication skills required for challenging situations such as breaking bad news. Given the increasing diversity in languages spoken in many countries the use of bilingual nurses as interpreters requires further consideration including the possibility of providing ‘interpreter training to willing bilingual clinicians who are formally determined as having high levels of fluency’ (Chang et al. 2019).

### 8.5 Study Limitations

Our review has several limitations. First, we restricted studies to those undertaken in adult acute care settings, and so the findings may not apply to paediatric or primary care settings. Second, we limited the review to studies where the experiences of nurses could be clearly extracted from the findings. Several studies reported the experiences of mixed samples of health professionals, and it is possible that if the experiences of nurses in all of these studies were available, the findings may have been different. Third, this review only included peer‐reviewed studies published in the English language, which again may have influenced the findings. Future reviews should consider including studies published in languages other than English. Nevertheless, this review has included studies from a number of countries where English is not the first language, and high rates of immigration have resulted in linguistically diverse populations, suggesting the findings from this review are generalisable.

## 9 Conclusion

This review sought to identify nurses' experiences of and preferences for working with professional HCIs when caring for patients with LEP in acute adult healthcare settings. While the review identified nurses' experiences of working with healthcare interpreters were overwhelmingly positive, concerns about the accuracy of the interpretation, unreliable technology, and at times being sidelined were evident. This review also identified that nurses commonly resorted to using bilingual nurse colleagues and family members when interpreters were unavailable, particularly for less complex care including fundamental care.

These findings contribute to our understanding of the challenges in caring for patients with LEP and the need to explore additional strategies to ensure nurses are able to safely communicate with patients, whether this is fundamental or more complex care. Future research should explore the decision‐making processes of nurses when determining the use of HCIs for complex versus simple nursing care, focusing on factors that influence these decisions and the outcomes for patient care and communication. This will require easy and timely access to interpreting services, whether this is in person, telephonic or video. A review of policy is also warranted to accommodate the use of bilingual healthcare professionals who have been assessed as language proficient (The Joint Commission 2017) when professional HCIs are not available or when nurses assess the situation as not requiring a professional HCI.

Finally, this review highlights the need for nurses to receive formal training on how to work collaboratively with healthcare interpreters to ensure patient centredness during three‐way conversations and encourage more patient involvement.

## Author Contributions

M.S., T.N., J.M., K.F., V.T. and B.E. were responsible for the study conception and design, organised the data collection, and performed the data analysis. M.S., T.N., J.M., K.F., V.T. and B.E. were responsible for drafting the manuscript. M.S., T.N., J.M., K.F., V.T. and B.E. made critical revisions to the paper for important intellectual content.

## Conflicts of Interest

The authors declare no conflicts of interest.

## Supporting information


Data S1.

Data S2.

## Data Availability

The data that support the findings of this study are available on request from the corresponding author. The data are not publicly available due to privacy or ethical restrictions.
