# Supplementary material for: Nurses' Experiences of Working With Healthcare Interpreters When Caring for Patients With Limited Proficiency in the Primary Language: A Qualitative Systematic Review
Source: J Clin Nurs. 2025 May 25;34(11):4874–98. doi: 10.1111/jocn.17740 (PMC12489433; doi:10.1111/jocn.17740)
Supplement: Supplementary file 1 — Data S1. [file JOCN-34-4874-s001.docx]

CINAHL Plus with Full Text

| S16 | S4 AND S8 AND S15 |
| --- | --- |
| S15 | S9 OR S10 OR S11 OR S12 OR S13 OR S14 |
| S14 | language n3 barriers |
| S13 | (MH "Communication Barriers") |
| S12 | ((non or limited) n3 english n6 patients) |
| S11 | (MH "Multilingualism") |
| S10 | (MH "English as a Second Language") (migrant* or immigrant or "non english speaking" or CALD or "culturally and linguistically diverse" or "limited english" or "english proficiency" or "english as a second") |
| S9 | (MH "Cultural Competence") OR (MH "Cultural Diversity") OR (MH "Acculturation") OR (MH "Cultural Values") OR (MH "Cultural Safety") |
| S8 | S5 OR S6 OR S7 |
| S7 | ((Health* or medical) n3 interpret*) |
| S6 | (MH "Translations") |
| S5 | (MH "Interpreter Services") OR interpret* |
| S4 | S1 OR S2 OR S3 |
| S3 | Transcultural Nursing/ |
| S2 | (MH "Nurse-Patient Relations") |
| S1 | (MH "Nurse Attitudes") OR (MH "Nurses+") |

Ovid MEDLINE

| 1 | nurs*.ti,ab,kw. |
| --- | --- |
| 2 | exp nurse/ or exp nursing/ or exp nurse patient relationship/ or exp nursing staff/ |
| 3 | Transcultural Nursing/ |
| 4 | interpret*.ti,ab,kw. |
| 5 | (translation or translating).ti,ab,kw. |
| 6 | ((Health$ or medical) adj3 interpret$).mp. |
| 7 | Translating/ or Communication Barriers/ |
| 8 | (migrant* or immigrant or "non english speaking" or CALD or "culturally and linguistically diverse" or "limited english" or "english proficiency" or "english as a second").mp. |
| 9 | ((non or limited) adj3 english adj6 patients).mp. |
| 10 | 1 or 2 or 3 |
| 11 | 4 or 5 or 6 or 7 |
| 12 | 8 or 9 |
| 13 | 10 and 11 and 12 |
